# Automatic Item Generation Measurement Models Respecting the Stochastic Sampling Space for Cross-Classified and Two-Level Sampling of Subjects and Incidentals

**DOI:** 10.1177/01466216261453386

**Published:** 2026-05-20

**Authors:** Philipp Jahn, David Jendryczko, Fridtjof W. Nussbeck

**Affiliations:** 1 Department of Psychology, 240145 University of Konstanz, Konstanz, Germany

**Keywords:** automatic item generation, cross-classified, figural memory, stochastic sampling space, structural equation modeling, two-level

## Abstract

In Automatic Item Generation (AIG), *item incidentals* refer to surface characteristics of an item that are assumed not to influence item parameters (e.g., item difficulty), whereas *item radicals* refer to attributes that are presumed to affect these parameters. Within the empirical validation process of the item generator, subjects and incidentals may either be sampled independently so that every subject sees every incidental (cross-classified sampling) for a radical, or incidentals may be sampled within each subject so that every subject only sees a specific set of incidentals (two-level sampling) for a radical. We present an approach for scrutinizing the effect of item incidentals relying on two classical test theory models that adhere to the stochastic sampling space of cross-classified and two-level sampling, respectively. We show how these may be used in combination to enable a more optimized investigation of incidental-induced variance within the item generator. We illustrate the approach with the figural short-term memory item-generator “figumem.” Results show that incidentals have little effect on item difficulty in the cross-classified model/sample and that the model parameters generalize to a larger set of incidentals in the two-level model/sample. Implications, limitations, and future research are discussed.

## Introduction

Automatic Item Generation (AIG) is a modern computer-based approach to test construction based on psychometric and cognitive models (Gierl & Lai, 2012). Usually, AIG consists of two steps: (1) item models are created that serve the role of templates or prototypes and (2) an algorithm manipulates specific elements of these item models to create the individual items (Gierl & Lai, 2012). Irvine (2013) introduced the terms *radicals* and *incidentals* for the specific elements to be varied in the second step. Radicals are those structural elements that influence item parameters like difficulties. In contrast, incidentals are surface elements that have no significant effect on the item parameters. For example, one could think of a test measuring numerical short-term memory (STM) with participants/subjects having to memorize a list of natural numbers with all numbers being of the same digit length (e.g., a list of two-digit numbers like 46, 73, and 91; or a list of three-digit numbers like 135, 957, and 641). Here, the digit length for each number would be the radical, since it is assumed to determine item difficulty (it is more difficult to remember a list of three-digit numbers than an equally long list of two-digit numbers). The exact numbers and their order within the list would be the incidentals, assuming that they do not determine item parameters (which might require some restrictions, e.g., excluding easily memorable repdigits like 11 or 222).

AIG can follow either a “strong” or “weak theory” approach. In the “strong theory” approach, the creation of item models is guided by scientific theories that can explain the cognitive features relevant for item responding and thus the item parameters (Gierl & Lai, 2012). On the other hand, in a “weak theory” approach, existing or newly created items require extensive field or pilot testing to determine the elements that influence the item parameters (Gierl & Lai, 2012). Since only radicals have a significant influence on item parameters (in contrast to incidentals), an item with a specific set of radical realizations can function as a so-called *parent item* (Glas et al., 2009). By letting incidentals vary randomly, *item families* consisting of parallel items with the same item parameters can be created (Irvine, 2013). The set of items that have the same radical realizations but different incidental realizations and therefore belong to one item family are commonly called *isomorphs* (Bejar, 2013) or *clones* (Arendasy et al., 2008).

In the past, AIG has primarily been used to develop items intended to measure cognitive abilities (Jendryczko et al., 2020). In addition to its use to measure knowledge of a specific subject (e.g., Circi et al., 2023; Falcão et al., 2022; Kurdi et al., 2020), AIG was predominantly used to create test material for figural (e.g., M. Arendasy & Sommer, 2005, 2010; Bejar, 1990; Bertling, 2012; Blum et al., 2016; Embretson, 2013; Gierl et al., 2008; Loe, 2019; Zeuch et al., 2011), verbal (e.g., M. Arendasy et al., 2006; M. E. Arendasy & Sommer, 2012; Bertling, 2012; Gierl et al., 2008; Holling et al., 2009; Loe, 2019; Loe et al., 2018) or numerical reasoning (e.g., Gierl et al., 2008; Loe, 2019) abilities. Driven by recent advances in machine learning (ML), particularly in deep learning (DL), natural language processing (NLP), and large language models (LLMs), research on AIG has increased substantially (e.g., Kıyak et al., 2024; Lee et al., 2024; von Davier, 2018). Recent developments enable the use of AIG for noncognitive constructs such as personality traits (Hommel et al., 2022) or even novel constructs (Götz et al., 2023) in a weak theory approach. Although AIG based on LLMs facilitates the generation of extensive initial item pools, human expertise remains essential for selecting promising items and conducting their validation.

In a strong theory approach, on the other hand, researchers have to identify the *construct representation* (Whitely, 1983), that is, the cognitive features that influence item responding (Harrison et al., 2017). Hypotheses about the relationship between structural features of items and their item parameters can be derived from the construct representation and tested with empirical data (Harrison et al., 2017). If the hypothesized relationship holds, this is evidence for the construct validity of the test and, thus, for newly created items with the same AIG process (Harrison et al., 2017). Since AIG is usually based on these quality control mechanisms, one strength of AIG is the possibility for decreased bias caused by misinterpretation in human item writing and the interpretation of test scores (Lai et al., 2009). AIG can help in creating a larger set of parallel test forms, which are, for example, useful when conducting longitudinal studies to avoid confounding effects of repeated item exposure (Jendryczko et al., 2019; Reeve & Lam, 2005). Even in non-longitudinal contexts, item exposure can be a problem if items are already known to the public (see Jendryczko et al., 2020; for a general overview of AIG-benefits, see Bertling, 2012).

As in traditional test construction, items created with an AIG procedure should be validated. According to Messick (1989), validity is “an integrated evaluative judgment of the degree to which empirical evidence and theoretical rationales support the adequacy and appropriateness of inferences and actions based on test scores.” Hence, the validation process is specific to the measure and the underlying theory and there is no simple “recipe” for all validation processes. This is also reflected in the conceptualization of validity as multidimensional, differentiating between different aspects of validity (e.g., Cooper, 2023). Modern approaches to test validation like the Evidence-Centered Design (ECD) framework build upon this multidimensional conceptualization and outline processes to establish validity by explicitly connecting task performance to actual ability (e.g., Mislevy & Haertel, 2006). Importantly, a test has to be reliable in order to be valid. That is, the impact of measurement error should be as small as possible. Thus, the investigation of test reliability can be considered an early step in the validation-process that is concerned with the nomothetic span (statistical relationships between test items) and not yet with the construct representation. In the reliability context, incidentals inherit an important meaning as they should not only be irrelevant for the between-person variability of the construct but should also not increase measurement-error variance. Put another way, while incidentals technically reflect a potential source of variance as they are known properties of an AIG-based test, different incidental realizations should have low impact and not affect measurement residuals. We refer to this concept as “noise stability.”

There are two options to investigate noise stability of automated item generators. Borrowing terminology from multilevel modeling, we will refer to them as the *cross-classified* and the *two-level* approach. In the cross-classified approach, the same incidentals are sampled for each subject (person/participant). This means that a certain number of items will be generated by the AIG procedure and presented to each subject. Hence, incidentals are nested in subjects, but subjects are also nested in incidentals (subjects and incidentals form two separate strata at level – 2 and the subject-incidental interaction locates at level – 1). This differs from the two-level approach, in which different incidentals are sampled for each subject. In consequence, each subject is presented with a unique set of items, with incidentals (level – 1) being nested in subjects (level – 2). Using the two-level approach comes with the advantage of allowing for better generalizations because the sample of used incidentals is larger than in the cross-classified approach (under the practical assumption that when comparing the two-level approach and the cross-classified approach, the number of presented items per subject in both approaches is the same). Therefore, inductive inference from the sample of realized items to the population of all possible AIG items is more trustworthy in the two-level approach. On the other hand, when using the two-level approach, each specific realization of incidentals is used for exactly one measurement. For this reason, the incidental effect on the item score cannot be estimated since it is not possible to disentangle it from the subject-incidental interaction. This, however, is possible in the cross-classified approach, where each realization of incidentals is presented to all subjects.

If the AIG procedure is properly implemented in terms of the realized incidentals, then noise stability will be observed across the two approaches. The proposed statistical structure at the person level should remain unaffected by the approach chosen, the between-incidental variance in the cross-classified approach should be marginally low, and the subject-incidental interaction variance should be approximately the same across the two approaches.

The aim of the current contribution is to show how to properly respect the stochastic sampling procedure in the statistical model for both study designs (cross-classified and two-level sampling). This will be illustrated with the figural STM-AIG test “figumem” created by Jendryczko et al. (2020). Parameter interpretations and procedures for drawing valid conclusions about the noise stability of an item generator are outlined, and the noise stability of figumem AIG is empirically evaluated.

The remainder of this paper is structured as follows: Figumem AIG and its theoretical assumptions will be presented. This includes describing what the radicals and what the incidentals are. Then, a general AIG Structural Equation Modelling (SEM)-framework for the cross-classified sampling will be derived and it will be shown how a simplified form applies to two-level sampling. Both models will be applied to empirical data sets. The paper concludes with a substantive discussion of the results, implications, limitations, and future research.

## Description and Brief Presentation of Figumem Items

Figumem is a test to assess figural-visual STM capacity (see Jendryczko et al., 2020 for its introduction and a user’s manual). It is based on AIG and is provided as an *R*-package.^1^ AIG of figumem items builds on a “strong theory” about the relationship between visual load and feature bindings. It is known that the number of units of information that can be remembered for a short period of time (the STM capacity) is severely constraint (Trapp et al., 2021). Regarding visual material, the number of units is not only referring to the number of visual objects, but also their visual (information) load. Visual load is operationalized as the time to find an object in a set of similar objects (Alvarez & Cavanagh, 2004). Visual load depends mostly on the complexity of bindings of stimulus features as shown in a series of experiments (e.g., color, shape, orientation, pattern; Ueno et al., 2010; 2011). Through manipulation of feature bindings of the presented objects, AIG of figumem items varies the visual load and thus the expected relative number of objects that can be stored in the STM rendering the items more or less difficult.

**Figure 1.**
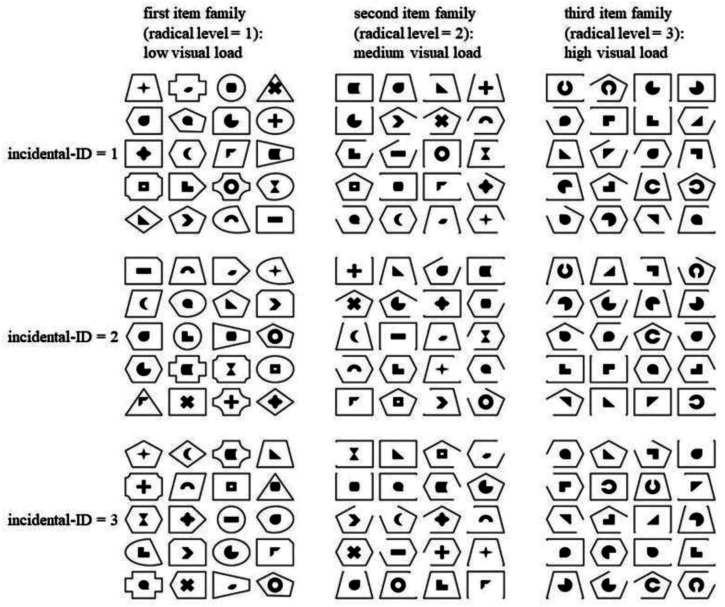
Examples of figumem items with different radical and incidental levels. Figure taken with permission from Jendryczko et al. (2020)

In the figumem test, subjects have to memorize which emblems (figures of set *A*) are associated with which frames (figures of set *B*; see Figure 1). Each item consists of 20 emblems, each surrounded by a frame. In the learning phase, the framed emblems are displayed in a 5 × 4 matrix for 1 minute. In the recall phase, the same emblems are displayed in a different order, yet the emblems are presented together with four different frames underneath them (1 correct, 3 distractors). The number of correctly marked frames represents the subjects’ score on the item (ranging from 0 to 20). While the *figumem* package also supports lower numbers of emblems, different time lengths of the learning phase, and an open response format, this paper is concerned only with the standard setup used in the original study by Jendryczko et al. (2020).

Based on theories of visual load and feature binding (Alvarez & Cavanagh, 2004; Ueno et al., 2010, 2011), a radical with three distinct levels is specified, resulting in the generation of three parent items. By varying the incidental realizations, three item families are created. For the first radical level, 20 distinct emblems and 20 distinct frames are used which differ only in their shape. Thus, the figures possess a low visual load as subjects only need to keep track of one stimulus feature (the shape). Therefore, the item difficulty should be low (examples can be seen in the first column of Figure 1). For the second radical level, the 20 distinct emblems from the first radical level are used again. Four frames from the first radical level (rectangle, trapeze, pentagon & hexagon) are modified such that four variants of each of them are created, each by erasing a different line of the corresponding frame. Those four frames and their variants are used for items with the second radical level. This increases the visual load compared to the first radical level since the frames are more similar and subjects have to keep track of two stimulus features: shape and completeness. Thus, item difficulty should be higher than for the first radical level (examples can be seen in the second column of Figure 1). For the third radical level, the frames from the second radical level are used again. Five emblems from the first radical level (right triangle, black circle with the missing quadrant in the top right, black circle with the prong in the top right, “L”-shape & black ring) are modified such that four variants of each of them are created that are being used instead of the original emblems. This is done for the black ring by editing out a piece of the ring at either the top, right side, bottom, or left side. The other four of the five emblems are rotated clockwise by either 0° (i.e., not changed at all), 90°, 180° or 270°. This increases the visual load compared to the first two radical levels since the frames and emblems are, respectively, more similar and subjects have to keep track of three stimulus features: shape, completeness and orientation. Thus, the item difficulty should be the highest of the three radical levels (examples can be seen in the third column of Figure 1).

While the three postulated radical levels determine three item families with theoretically distinct item difficulties, there are three different postulated incidentals that determine specific items. It is randomly varied (1) which emblem is surrounded by which frame, (2) in which order the figures in the 5 × 4 matrix are presented, and (3) which three frames are used as distractors (with the constraint that each frame appears equally often as a distractor).

The hypothesized psychometric properties were largely confirmed in an empirical study using the cross-classified approach (Jendryczko et al., 2020). Radical level mostly determined the item difficulties while incidentals had a small, yet not negligible effect. For items with low visual load, subjects were on average able to memorize roughly 10–11 figure associations with considerable variation. For example, the difference in predicted scores for two specific incidental realizations used in the study was 6.82% (an item score of 0.74). For items with medium visual load, the influence of incidentals was slightly higher (and also higher than for items with high visual load). For example, the difference was 12.36% (an item score of 1.11) when comparing two specific incidental realizations used in the study (subjects were on average able to memorize roughly 9-10 figure associations). Accordingly, Jendryczko et al. (2020) recommended taking these findings into account when administering the test and adopting a conservative stance when interpreting small differences in item scores. Despite these potential influences, *figumem* “holds the potential for an efficient, reliable, and repeatable assessment of figural memory in various non-clinical and clinical populations” (Jendryczko et al., 2020, p. 12).

## Model Development

We derive the underlying psychometric model relying on the example of figumem (see Figure 1). Yet, the model applies to all cases where subjects and incidentals are cross-classified. In the specific case of figumem, subjects are sampled from the population of subjects with *s* denoting the specific sampled subject and, additionally, surface characteristics of an item are sampled from the population of incidentals with *i* denoting the specific surface characteristic. If every subject “sees” every incidental realization (and vice versa) the sampling process is cross-classified (for a formal definition of the stochastic sampling space see Koch et al., 2016). Further, there is a non-random radical variable for which three structurally different outcomes are given (*r* = 1, 2, or 3) which correspond to item-difficulty.

**Figure 2.**
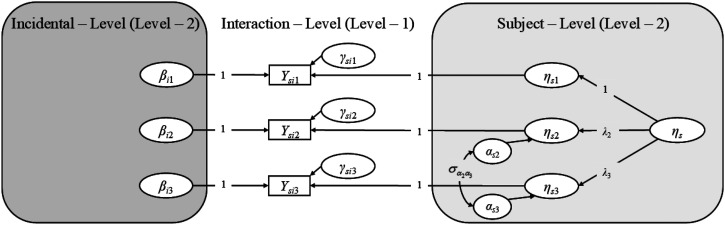
Example for the proposed classical test theory AIT-model for a construct measured with three different radical levels (*r* = 1, 2, or 3). $Y_{sir}$ = observed response variable of subject *s* to an item comprised of incidental realization *i* and radical level *r*, $\gamma_{sir}$ = subject-incidental interaction specific noise variable for radical level *r*, $\eta_{s}$ = latent subject-ability variable for the reference-radical level *r* = 1, $\lambda_{r}$ = factor-loading for the non-reference radical level *r* ≠ 1 on the latent ability, $\alpha_{sr}$ = radical-effect variable for the non-reference radical level *r* ≠ 1 at the subject-level, $\sigma_{\alpha_{2}\alpha_{3}}$ = radical-effect covariance for the two non-reference radical levels *r* = 2 and *r* = 3 at the subject level, $\beta_{ir}$ = incidental-effect variable for radical level *r*. Intercepts are not shown. If incidentals are not sampled independently of subjects but nested within subjects (two-level sampling process instead of cross-classified sampling process), the incidental level drops from the model and the $\beta_{ir}$ are confounded within the noise variables at level – 1

In the following, the cross-classified model will be formally defined step by step. It will be shown how the model for the two-level sampling process (each subject only “sees” her or his individual set of incidentals) can be derived by simplifying the cross-classified model. The models are shown in Figure 2.

According to classical test theory (CTT), any observed response $Y_{sir}$ of subject *s* to incidental realization *i* for radical level *r* can be decomposed into a true score $\tau_{sir}$ and a measurement-error $\varepsilon_{sir}$ :

(1) $$Y_{sir}=\tau_{sir}+\varepsilon_{sir}.$$

The true score can be further decomposed into expected values according to the stochastic sampling space (Jendryczko & Nussbeck, 2024; Koch et al., 2016):

(2) $$\tau_{sir}=\mu_{r}+\eta_{sr}+\beta_{ir}+\psi_{sir}.$$

Here, the unconditional expectation for the true score $E\left(\tau_{sir}\right)$ is given by the intercept (mean) for radical level *r*: $\mu_{r}$ . The conditionally expected deviation from $\mu_{r}$ given the outcome *s* of the random subject variable $E\left(\tau_{sir}‐\mu_{r}|s\right)$ for radical level *r* is denoted as $\eta_{sr}$ and can be interpretated as the latent subject-ability variable for radical level *r*. The conditionally expected deviation from $\mu_{r}$ given the outcome *i* of the random incidental variable $E\left(\tau_{sir}‐\mu_{r}|i\right)$ for radical level *r* is denoted as $\beta_{ir}$ and can be interpretated as a latent incidental effect. Lastly, the conditionally expected residual deviation from the previous expectations given the specific combination of *s* and *i* (the latent subject-incidental interaction effect) is denoted as $E\left(\tau_{sir}‐\left(\mu_{r}+\eta_{sr}+\beta_{ir}\right)|s,i\right)=\psi_{sir}$ .

The ability variables for different radical levels are assumed to be correlated since they are assumed to measure the same psychological construct within subjects. We can, thus, define the latent ability of one reference-radical level (e.g., *r* = 1) as a standard ( $\eta_{s1}=\eta_{s}$ ) and regress the abilities for the non-reference radical levels (*r* ≠ 1) on this standard ability measure:

(3) $$\eta_{sr}=\lambda_{r}\eta_{s}+\alpha_{sr}.$$

Here, $\alpha_{sr}$ denotes a non-reference radical level specific residual given the reference-radical level $E\left(\eta_{sr}|\eta_{s}\right)=\lambda_{r}\eta_{s}$ and can be interpreted as a latent radical effect that goes beyond item difficulty represented by the unconditional expectation *µ*_*r*_. It reflects a specific interplay between a subject’s ability and the non-reference radical level. Note that no intercept is given in equation (3) since the latent ability variables reflect conditional deviations and, therefore, have unconditional expectations of zero.

Putting everything together, we arrive at

(4) $$Y_{si1}=\mu_{1}+\eta_{s}+\beta_{i1}+\psi_{si1}+\varepsilon_{si1}$$

for the reference radical level *r* = 1 and

(5) $$Y_{sir}=\mu_{r}+\lambda_{r}\eta_{s}+\alpha_{sr}+\beta_{ir}+\psi_{sir}+\varepsilon_{sir}$$

for the non-reference radical levels *r* ≠ 1. Importantly, no incidental is repeated across different radical levels which means that the different $\beta_{ir}$ and $\psi_{sir}$ across different radical levels are, respectively, uncorrelated. Thus, no measurement structure can be assumed for these latent variables. This also implies that, empirically, the latent subject-incidental interaction effect cannot be disentangled from measurement error, giving

(6) $$\begin{array}{l} Y_{si1}=\mu_{1}+\eta_{s}+\beta_{i1}+\gamma_{si1}\;\text{and}\\ Y_{sir}=\mu_{r}+\lambda_{r}\eta_{s}+\alpha_{sr}+\beta_{ir}+\gamma_{sir\;} \end{array}$$

with $\gamma_{si1}=\psi_{si1}+\varepsilon_{si1}$ and $\gamma_{sir}=\psi_{sir}+\varepsilon_{sir}$ . Note that, from a practical viewpoint, this confound may be regarded to be of little importance: γ may be considered a random noise variable since there should not be any systematics behind the subject-incidental interaction. Note further that different radical-effect variables (*r* ≠ *r’*) may covary. These covariances will be denoted as $\sigma_{\alpha_{r}\alpha_{r^{\prime }}}$ in the following (the respective correlation will be denoted as $\rho_{\alpha_{r}\alpha_{r^{\prime }}}$ ).

In the case of a cross-classified sampling procedure (incidentals are repeated across subjects), the model as formulated in equation (6) is well-constrained (i.e., “identified” in frequentist terminology) and estimable as a structural equation model (SEM). In the case of a two-level sampling procedure, incidental effects $\beta_{ir}$ cannot be estimated as every incidental realization only appears once (is only seen by one subject). Any potential incidental effects are then additionally confounded within the noise variable γ and the model reduces to:

(7) $$\begin{array}{l} Y_{si1}=\mu_{1}+\eta_{s}+\gamma_{si1}\;\text{an}d\\ {\;Y}_{sir}=\mu_{r}+\lambda_{r}\eta_{s}+\alpha_{sr}+\gamma_{sir.\;} \end{array}$$

If incidentals have no or only negligible influence, then: (1) Any variability at the person-level should remain stable regardless of the employed incidentals; (2) the variability of the incidental effect *β*_*ir*_ around the mean of zero should be zero or very low in the cross-classified approach; and (3) the variability of the random noise variable *γ*_*sir*_ should be stable across the two approaches. As variances cannot easily be interpreted, one should also investigate variance components in addition to absolute variances.

## Variance Decomposition and Consistency Parameters

Since all latent variables manifesting in an observation are orthogonal (see Figure 2) the variance of each observed variable equals the sum of the latent variances weighted by the factor loadings:

(8) $$\sigma_{Y_{sir}}^{2}=\lambda_{r}^{2}\sigma_{\eta_{s}}^{2}+\sigma_{\alpha_{r}}^{2}+\sigma_{\beta_{r}}^{2}+\sigma_{\gamma_{r}}^{2}$$

Therefore, we can define three meaningful consistency-parameters (see also Koch et al., 2016) for the subject-level variance as relative variance components. The radical-level consistency coefficient (*RCon*_*r*_) for a specific non-standard radical level (*r* ≠ 1) is defined as:

(9) $$RCon_{r}=\lambda_{r}^{2}\sigma_{\eta_{s}}^{2}/\left(\lambda_{r}^{2}\sigma_{\eta_{s}}^{2}+\sigma_{\alpha_{r}}^{2}\right).$$

It depicts the consistency with which the ability variability as assessed with the standard radical level (*r* = 1) manifests in the ability variability as assessed with a non-standard radical level (*r* ≠ 1) and, thus, the degree to which the construct remains stable across radical levels. If different AIG-items measure the exact same construct with only different levels of difficulty, radical-level consistency should be close to one as mere differences in difficulty across items should be fully captured by different unconditional expectations (different $\mu_{r}$ ).

The Level-2 consistency coefficient for a specific radical level *r* (*L*2*Con*_*r*_) depicts the subject-level variance relative to the total variance at level-2, that is, the variance at the subject level plus the variance at the incidental level:

(10) $$L2Con_{r}=\left(\lambda_{r}^{2}\sigma_{\eta_{s}}^{2}+\sigma_{\alpha_{r}}^{2}\right)/\left(\lambda_{r}^{2}\sigma_{\eta_{s}}^{2}+\sigma_{\alpha_{r}}^{2}+\sigma_{\beta_{r}}^{2}\right).$$

This consistency should also be close to one if the AIG procedure works sufficiently with regard to noise stability, as it is assumed that different incidental realizations should not cause additional variability ( $\sigma_{\beta_{r}}^{2}$ should be low).

The Level-1 consistency coefficient for a specific radical level *r* (*L*1*Con*_*r*_) shows the subject-level variance relative to the total model-implied variance of the observed variable:

(11) $$L1Con_{r}=\left(\lambda_{r}^{2}\sigma_{\eta_{s}}^{2}+\sigma_{\alpha_{r}}^{2}\right)/\sigma_{Y_{sir}}^{2}=\left(\lambda_{r}^{2}\sigma_{\eta_{s}}^{2}+\sigma_{\alpha_{r}}^{2}\right)/\left(\lambda_{r}^{2}\sigma_{\eta_{s}}^{2}+\sigma_{\alpha_{r}}^{2}+\sigma_{\beta_{r}}^{2}+\sigma_{\gamma_{r}}^{2}\right).$$

This consistency should be close to one if incidental-specific variability ( $\sigma_{\beta_{r}}^{2}$ ) is low and random noise ( $\sigma_{\gamma_{r}}^{2}$ ) is low. Moreover, even in the case of substantial random noise, the consistency coefficient should be stable across sampling procedures if random-noise variability ( $\sigma_{\gamma_{r}}^{2}$ ) does not substantially depend on the concrete realizations of incidentals. Note that $\lambda_{1}=\lambda_{1}^{2}=1$ and $\sigma_{\alpha_{1}}^{2}=0$ for the reference-radical level *r* = 1. Note further that in the case of a two-level sampling process, $\sigma_{\beta_{r}}^{2}=0$ since no incidental effects $\beta_{ir}$ are modeled which means that *L*2*Con*_*r*_ is not meaningful and that *L*1*Con*_*r*_ simplifies to:

(12) $$L1Con_{r}=\left(\lambda_{r}^{2}\sigma_{\eta_{s}}^{2}+\sigma_{\alpha_{r}}^{2}\right)/\sigma_{Y_{sir}}^{2}=\left(\lambda_{r}^{2}\sigma_{\eta_{s}}^{2}+\sigma_{\alpha_{r}}^{2}\right)/\left(\lambda_{r}^{2}\sigma_{\eta_{s}}^{2}+\sigma_{\alpha_{r}}^{2}+\sigma_{\gamma_{r}}^{2}\right)$$

In the following, two empirical examples will be presented. Data and code for both examples can be found in the supplementary materials at https://osf.io/ern58/.

## Empirical Example: Data From the Cross-Classified Sampling Process

We start with a summary of the data-collection process described in the original paper by Jendryczko et al. (2020). Data collected from this study will be used to illustrate the cross-classified model (equation (6)).

### Sample

The final sample consisted of *N* = 208 subjects (146 female, 61 male and 1 not stated). On average, subjects were 22.51 years old (*SD* = 6.18) with most of them being university students. Detailed information about the sample is given in Jendryczko et al. (2020).

### Materials

Nine different figumem items (as displayed in Figure 1) were used (three of each radical level, all consisting of 20 emblem-frame pairings). After the learning phase (60 seconds), the emblems were displayed one below the other and subjects had to give their responses and scroll down to the next emblem. Each emblem was presented together with four frames (one correct option and three distractors; presented below each emblem).

### Procedure

After giving informed consent and receiving the instruction not to use supporting devices (like paper and pencil), subjects were presented with a black rectangle of the size of a figumem item to check if the items fit on the subjects’ screens. Figumem items were explained with the presentation of an example item for each radical level and a practice item that was presented on the next page. The practice item contained only two framed emblems. It was the only item where subjects received feedback and could click a “next” button if they were able to memorize the two emblem-frame pairings before the end of the learning phase. If they were not able to memorize both pairings correctly a second practice item was presented.

**Figure 3.**
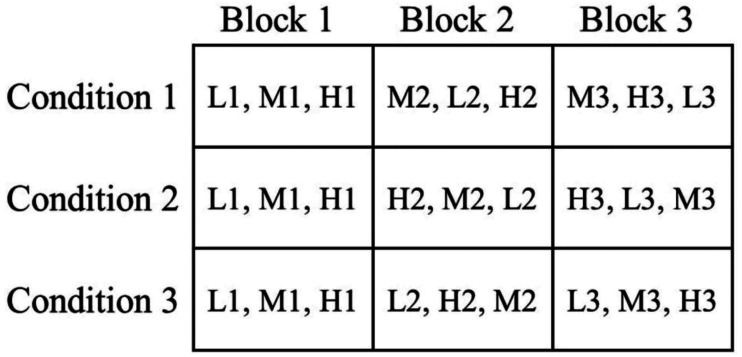
Latin Square for item order in both studies. Subjects were (pseudo) randomized to one of the three conditions and went through the three blocks in the same order. Each block contains one item of each radical level (L = low visual load, M = medium visual load, H = high visual load). This Latin Square was used in the original study by Jendryczko et al. (2020) and in the new study using the two-level approach. In the original study, the number behind each letter (that indicates the radical level) signifies the incidental-ID of a specific item. In the new validation study presented in this paper in which each subject responds to a distinct set of items, the number behind each letter (that indicates the radical level) signifies a block-ID of a specific item. This block-ID only indicates in which block the distinct item is presented. Figure taken with permission from Jendryczko et al. (2020)

Thereafter, the nine figumem items were presented to the subjects. The order of presentation was varied to deal with confounding sequence effects. To avoid negatively affecting subject motivation and performance by starting the survey with multiple difficult items, a Latin Square design (e.g., Jacobson & Matthews, 1996) was used to determine groups of presentation order instead of a full randomization of item sequence (see Figure 3). Items were distributed into three blocks, each of them containing one item of each radical level. Subjects were (pseudo) randomly assigned to one of three conditions (while targeting an equal distribution). The first block constituted a warm-up phase in which the theoretically easiest item (low visual load) is presented first, then the item of theoretically medium difficulty (medium visual load) and, last, the item of the theoretically highest difficulty (high visual load). This sequence of the first block was the same for all three conditions. In the other two blocks, the sequence of items was rotated according to the Latin Square design, that is, an item of each radical level appeared once at each position. All subjects went through the blocks in the same order. A forced-choice design was chosen to avoid missing data. After responding to all nine items, socio-demographic information was collected.

## Empirical Example: Data From the Two-Level Sampling Process

In the following, a new empirical study using two-level sampling will be presented. This data will be used for an illustration of the two-level model (equation (7)). The Ethics Committee (institutional review board, IRB) of the University of Konstanz waived the requirement for approval of the study. IRB-number: IRB25KN009-03/w.

### Sample

Subjects were recruited via the online platform SONA (Sona Systems, Ltd, 2022) mainly from the University of Konstanz. Out of the 239 subjects, 25 had to be excluded because they indicated that their screen did not always fit the complete figumem item. Five subjects reported problems with image loading. Six subjects did not solve the practice item consisting of only two emblem-frame pairings on their second try. Thus, the final sample consisted of *N* = 204 subjects, with one subject having produced a missing value due to a technical error on one item for the second radical level. 169 subjects were female, 31 were male and four subjects selected the gender option “diverse.” On average, subjects were 22.25 years old (*SD* = 5.04). Most subjects were university students studying Psychology. When asked about their highest degree, 175 subjects responded high school diploma (“A-level”), 14 subjects had a Bachelor’s degree, nine subjects had a Master’s degree or an equivalent degree (“Diplom”), 1 subject responded “Secondary School” and five subjects chose “other” and indicated they had either completed vocational training or obtained a degree equivalent to A-levels but from another country. Psychology students were able to obtain course credits for participation. There was no compensation for other subjects.

### Materials

The study was created using the online survey platform FiguGen (Jahn, 2023) which supports online figumem AIG with the R-package by Jendryczko et al. (2020). Three figumem items were used to describe the task to subjects. In the testing phase, nine distinct items were created “on-the-fly” for each subject (three items of each radical level, all consisting of 20 emblem-frame pairings). After the learning phase, the emblems were displayed one emblem at each page and subjects had to give their responses and could go to the next (or to the previous) emblem by using the “next” (the “back”) button. Below each emblem four frames were presented (1 correct, 3 distractors). Subjects were prevented by FiguGen from using the browser’s “back” button to avoid letting them see the image to be memorized more than once. Another figumem item with only two framed emblems was used as a practice item.

### Procedure

The procedure was the same as in the original study by Jendryczko et al. (2020) except for the fact that no item was presented to more than one subject. Again, a Latin Square Design was used to determine groups of presentation order to prevent confounding sequence effects as in Jendryczko et al. (2020; see Figure 3).

## Empirical Example: Analytic Strategy

We applied the AIG model to the cross-classified and the two-level data for figumem as previously described. The easiest radical condition (high dissimilarity for both inner emblems and outer shapes, respectively) was chosen as the reference-radical level. This choice is based on the rationale that the easiest radical level requires only the most “basic” memorization process connecting a specific emblem to a specific outer shape, whereas the other two radical conditions require additional disassociation processes to distinguish the outer shapes from each other (radical level 2) or to distinguish both the outer shapes from each other and the inner emblems from each other (radical level 3).

We estimated both models using Bayesian Markov-Chain-Monte-Carlo (MCMC) Gibbs sampling (three chains for each model) with mostly weakly informative priors (see Appendix A) in Mplus (version 8.7; Muthén & Muthén, 1998–2017). For both models, the number of chain iterations was set to 1,000,000 with the first half treated as warm-up (the estimation-time for the cross-classified model was 17 minutes and the estimation-time for the two-level model was 16 minutes on a 3.4 GHz processor personal computer). This procedure was chosen for two reasons: (1) To the best of our knowledge, maximum-likelihood estimation for cross-classified SEMs containing freely estimated factor loadings is yet to be derived (see Jeon & Rijmen, 2014). (2) Bayesian posterior sampling allows for the computation of credibility intervals for variance components, that is, the consistency coefficients displayed above. Model fit was investigated via the Bayesian posterior predictive checking procedure (BPPC; e.g., Asparouhov & Muthén, 2021; Gelman et al., 1996) using the $\chi^{2}$ -statistics. Better model fit is indicated by the closeness of the posterior predictive *p*-value to .5.

We additionally computed the posterior distributions of differences between the different unconditional expectations of radical levels ( $\mu_{r}‐\mu_{r^{\prime }}$ with *r* ≠ *r’*) within each model. This allows to investigate whether items with different radical levels differed significantly in their difficulty (indicated by a non-inclusion of zero in the credibility interval). Furthermore, we computed the posterior distributions of parameters that are identical across the two models, in order to investigate whether these parameters differed significantly across the samples. The data and analysis scripts can be found in the supplementary material.

## Empirical Example: Results and Discussion

**Figure 4.**
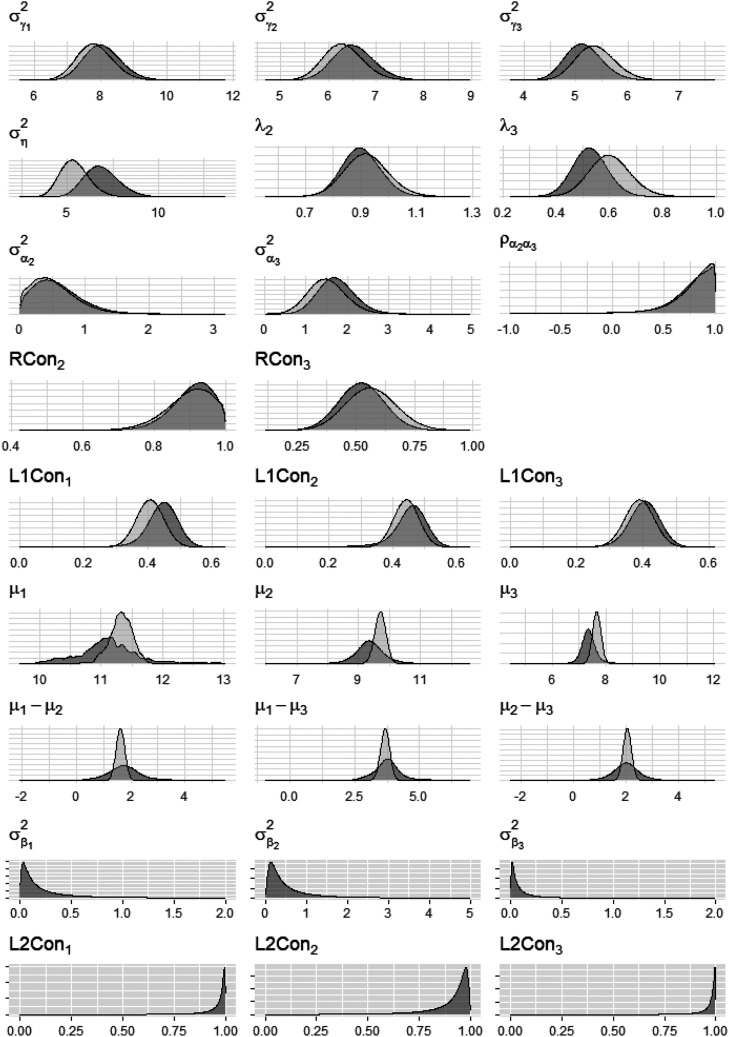
Posterior density distributions of model parameters. $\sigma_{\gamma_{r}}^{2}$ = subject-incidental interaction specific noise variance for radical level *r*, $\sigma_{\eta }^{2}$ = latent subject-ability variance for the reference-radical level *r* = 1, $\lambda_{r}$ = factor loading for the non-reference radical level *r* ≠ 1 on the latent ability η , $\sigma_{\alpha_{r}}^{2}$ = radical-effect variance for the non-reference radical level *r* ≠ 1 at the subject level, $\rho_{\alpha_{2}\alpha_{3}}$ = radical-effect correlation for the two non-reference radicals *r* = 2 and *r* = 3 at the subject level, $RCon_{r}$ = radical-level consistency of the standard radical level within a non-standard radical level (*r* ≠ 1), $L1Con_{r}$ = Level-1 consistency of the subject-level variance for radical level *r*, $\mu_{r}$ = intercept (mean) for radical level *r*, $\sigma_{\beta_{r}}^{2}$ = incidental-effect variance for radical level *r*, $L2Con_{r}$ = Level-2 consistency of the subject-level variance for radical level *r*. Distributions colored in dark-gray relate to the cross-classified model, distributions colored in light-gray relate to the two-level model. The plots in the last two rows relate to the parameters specific to the cross-classified model, which is why only dark-gray colored distributions are shown in these cases

According to the Gelman-Rubin criterion (Gelman & Rubin, 1992), both models converged with the highest potential scale reduction factors being below 1.1 at the final iteration (cross-classified model: 1.015, two-level model: 1.001). Yet, the posterior distribution plots (see Figure 4) and the trace plots (see supporting information) for $\mu_{1}$ indicated convergence problems for this parameter in both models. Thus, we will supplement any inferences concerning the intercepts of the models with *t*-tests.

The model applied to the cross-classified data yielded an excellent fit (*M*( $\Delta \chi^{2}$ ) = −0.793, 95%-confidence interval = [−16.069, 17.391], *p* = .459). The model applied to the two-level data yielded a less ideal but still acceptable fit (*M*( $\Delta \chi^{2}$ ) = −7.424, 95%-confidence interval = [−9.867, 24.520], *p* = .188).

Table 1 presents the parameter point-estimates (medians) and 95%-equal tailed credibility intervals (CI) for the parameters of the cross-classified and the two-level model respectively, as well as these statistics for the differences of the parameters between the models. Figure 4 shows the posterior distributions of the parameters graphically. As can be seen, the posterior-distributions overlap to a large degree, indicating no substantial differences in common model parameters between sampling procedures/groups. This is further corroborated by the fact that all CI for differences in parameters across models contain zero. In the following, the 95%-CI bounds are given in brackets.

**Table 1.** Point estimates and 95%-equal tailed credibility intervals [in brackets] for the parameters of the models and their difference between the models

Parameter	Cross-classified	Two-level	Difference
$\sigma_{\gamma_{1}}^{2}$	8.036 [7.037, 9.221]	7.813 [6.838, 8.963]	0.223 [−1.298, 1.757]
$\sigma_{\gamma_{2}}^{2}$	6.508 [5.742, 7.405]	6.292 [5.552, 7.159]	0.216 [−0.941, 1.378]
$\sigma_{\gamma_{3}}^{2}$	5.133 [4.496, 5.893]	5.365 [4.696, 6.165]	−0.232 [−1.254, 0.779]
$\sigma_{\eta }^{2}$	6.811 [5.173, 8.913]	5.339 [3.978, 7.102]	1.466 [−0.929, 3.970]
$\lambda_{2}$	0.898 [0.771, 1.040]	0.916 [0.770, 1.077]	−0.017 [−0.222, 0.185]
$\lambda_{3}$	0.525 [0.403, 0.656]	0.598 [0.456, 0.753]	−0.073 [−0.270, 0.121]
$\sigma_{\alpha_{2}}^{2}$	0.530 [0.057, 1.410]	0.474 [0.042, 1.302]	0.051 [−0.905, 1.050]
$\sigma_{\alpha_{3}}^{2}$	1.714 [0.933, 2.649]	1.492 [0.677, 2.446]	0.224 [−1.011, 1.462]
$\rho_{\alpha_{2}\alpha_{3}}$	0.814 [0.268, 0.991]	0.827 [0.214, 0.992]	−0.009 [−0.581, 0.619]
$RCon_{2}$	0.913 [0.774, 0.991]	0.905 [0.748, 0.991]	0.008 [−0.159, 0.184]
$RCon_{3}$	0.524 [0.339, 0.715]	0.562 [0.359, 0.779]	−0.039 [−0.323, 0.242]
$L1Con_{1}$	0.447 [0.329, 0.530]	0.406 [0.325, 0.489]	0.039 [−0.103, 0.157]
$L1Con_{2}$	0.459 [0.301, 0.541]	0.443 [0.365, 0.520]	0.015 [−0.155, 0.130]
$L1Con_{3}$	0.405 [0.301, 0.490]	0.390 [0.304, 0.474]	0.015 [−0.121, 0.136]
$\mu_{1}$	11.106 [10.171, 12.447]	11.331 [10.936, 11.665]	−0.207 [−1.153, 1.028]
$\mu_{2}$	9.358 [8.355, 10.508]	9.702 [9.336, 10.052]	−0.340 [−1.406, 0.853]
$\mu_{3}$	7.351 [6.830, 8.126]	7.655 [7.343, 7.960]	−0.300 [−0.906, 0.516]
$\mu_{1}‐\mu_{2}$	1.741 [0.379, 3.282]	1.624 [1.307, 1.935]	0.121 [−1.275, 1.691]
$\mu_{1}‐\mu_{3}$	3.746 [2.635, 5.080]	3.672 [3.312, 4.011]	0.078 [−1.072, 1.425]
$\mu_{2}‐\mu_{3}$	2.001 [0.770, 3.205]	2.046 [1.736, 2.358]	−0.046 [−1.309, 1.196]
$\sigma_{\beta_{1}}^{2}$	0.160 [0.010, 4.813]		
$\sigma_{\beta_{2}}^{2}$	0.400 [0.054, 7.146]		
$\sigma_{\beta_{3}}^{2}$	0.066 [0.006, 2.045]		
$L2Con_{1}$	0.977 [0.583, 0.999]		
$L2Con_{2}$	0.938 [0.457, 0.991]		
$L2Con_{3}$	0.982 [0.635, 0.998]		

*Notes*. $\sigma_{\gamma_{r}}^{2}$ = subject-incidental interaction specific noise variance for radical level *r*, $\sigma_{\eta }^{2}$ = latent subject-ability variance for the reference-radical level *r* = 1, $\lambda_{r}$ = factor loading for the non-reference radical level *r* ≠ 1 on the latent ability η , $\sigma_{\alpha_{r}}^{2}$ = radical-effect variance for the non-reference radical level *r* ≠ 1 at the subject level, $\rho_{\alpha_{2}\alpha_{3}}$ = radical-effect correlation for the two non-reference radical levels *r* = 2 and *r* = 3 at the subject level, $RCon_{r}$ = radical-level consistency of the standard radical level within a non-standard radical level (*r* ≠ 1), $L1Con_{r}$ = Level-1 consistency of the subject-level variance for radical level *r*, $\mu_{r}$ = intercept (mean) for radical level *r*, $\sigma_{\beta_{r}}^{2}$ = incidental-effect variance for radical level *r*, $L2Con_{r}$ = Level-2 consistency of the subject-level variance for radical level *r*.

As hypothesized, the easiest radical level was the first one, with an expectation of 11.106 [10.171, 12.447] correctly memorized associations in the cross-classified model and an expectation of 11.331 [10.936, 11.665] correctly memorized associations in the two-level model. The third radical level was the most difficult one with an expectation of 7.351 [6.830, 8.126] correctly remembered associations in the cross-classified model and 7.655 [7.343, 7.960] in the two-level model. The second radical level had intermediate difficulty with an expectation of 9.358 [8.355, 10.508] correctly remembered associations in the cross-classified model and 9.702 [9.336, 10.052] in the two-level model. Differences between these difficulties were significant in each model (cross-classified: $\mu_{1}‐\mu_{2}$ = 1.741 [0.379, 3.282]; $\mu_{1}‐\mu_{3}$ = 3.746 [2.635, 5.080]; $\mu_{2}‐\mu_{3}$ = 2.001 [0.770, 3.205]; two-level: $\mu_{1}‐\mu_{2}$ = 1.624 [1.307, 1.935]; $\mu_{1}‐\mu_{3}$ = 3.672 [3.312, 4.011]; $\mu_{2}‐\mu_{3}$ = 2.046 [1.736, 2.358]). Across the two models, intercepts and differences in intercepts were not significantly different within the Bayesian analysis. These findings were corroborated by the *t*-tests (see Table 2) with the only exception being the intercept for the third radical level. Here, the difference across the two models was significant according to the frequentist analysis ( $\mu_{3‐cross‐classified}‐\mu_{3‐two‐level}$ = −0.333, *t*(1,233.40) = −1.994, *p* = .046). Yet, it must be stated that the *t*-tests do not take the dependencies due to subjects into account so that confidence intervals are likely understated and this might reflect a type-1 error.

**Table 2.** Means of variables (and difference variables) in the cross-classified and the two-level samples (second and third column) and mean comparison across the samples (last column)

Variable	Cross-classified	Two-level	Difference
Y_si1_	11.083, *t* (623) = 71.445, *p* < .001	11.310, *t* (611) = 76.881, *p* < .001	−0.227, *t* (1,231.70) = −1.062, *p* = .288
Y_si2_	9.316, *t* (623) = 65.37, *p* < .001	9.687, *t* (610) = 71.545, *p* < .001	−0.371, *t* (1,231.00) = −1.891, *p* = .059
Y_si3_	7.304, *t* (623) = 62.288, *p* < .001	7.637, *t* (611) = 64.323, *p* < .001	−0.333, *t* (1,233.40) = −1.994, *p* = .046
Y_si1_ – Y_si2_	1.768, *t* (623) = 11.505, *p* < .001	1.627, *t* (610) = 11.032, *p* < .001	0.141, *t* (1,231.90) = 0.661, *p* = .509
Y_si1_ – Y_si3_	3.779, *t* (623) = 23.200, *p* < .001	3.673, *t* (611) = 22.558, *p* < .001	0.106, *t* (1,233.90) = 0.459, *p* = .647
Y_si2_ – Y_si3_	2.011, *t* (623) = 13.795, *p* < .001	2.047, *t* (610) = 14.594, *p* < .001	−0.036, *t* (1,232.00) = −0.180, *p* = .858

*Note. Y*_*sir*_ = observed response variable (*s* = subject, *i* = incidental realization, *r* = radical level). *t*-tests in the last column reflect two-sample Welch-tests without the homoscedasticity assumption. The remaining *t*-tests are one-sample *t*-tests.

The largest difference in model parameters between sampling procedures/groups was observed for the variance in latent ability assessed with the first radical level (cross-classified: 6.811 [5.173, 8.913]; two-level: 5.339 [3.978, 7.102]; difference: 1.466 [−0.929, 3.970]). Factor loadings were higher for the second radical level (cross-classified: 0.898 [0.771, 1.040]; two-level: 0.916 [0.770, 1.077]; difference: −0.017 [−0.222, 0.185]) than for the third radical level (cross-classified: 0.525 [0.403, 0.656]; two-level: 0.916 [0.770, 1.077]; difference: −0.073 [−0.270, 0.121]), while the residual radical-effect variance was lower for the second radical level (cross-classified: 0.530 [0.057, 1.410]; two-level: 0.474 [0.042, 1.302]; difference: 0.051 [−0.905, 1.050]) than for the third radical level (cross-classified: 1.714 [0.933, 2.649]; two-level: 1.492 [0.677, 2.446]; difference: 0.224 [−1.011, 1.462]). This yielded high radical consistency for the second radical level (cross-classified: 0.913 [0.774, 0.991]; two-level: 0.905 [0.748, 0.991]; difference: 0.008 [−0.159, 0.184]) and much lower radical consistency for the third radical level (cross-classified: 0.524 [0.339, 0.715]; two-level: 0.562 [0.359, 0.779]; difference: −0.039 [−0.323, 0.242]). Accordingly, we can conclude that the additional visual load due to varying completeness of the frames introduced only little additional variance between subjects (little additional differential cognitive operations) in comparison to visual load due to shape alone. However, visual load due to shape, completeness, and orientation introduced much more additional variance between subjects (much more additional differential cognitive operations) in comparison to visual load alone. The radical effects correlated strongly but this correlation was estimated with weak precision (cross-classified: 0.814 [0.268, 0.991]; two-level: 0.827 [0.214, 0.992]; difference: −0.009 [−0.581, 0.619]). Such a strong radical-effect correlation would indicate that the second radical level can explain much of the remaining variance in the third radical level after the variance of the first radical level has been partialled out.

In the cross-classified model, comparatively little variability due to incidental effects was found ( $\sigma_{\beta_{1}}^{2}$ = 0.160 [0.010, 4.813]; $\sigma_{\beta_{2}}^{2}$ = 0.400 [0.054, 7.146]; $\sigma_{\beta_{3}}^{2}$ = 0.066 [0.006, 2.045]), yielding high level – 2 consistency coefficients ( $L2Con_{1}$ = 0.977 [0.583, 0.999]; $L2Con_{2}$ = 0.938 [0.457, 0.991]; $L2Con_{3}$ = 0.982 [0.635, 0.998]).

In light of incidentals having such little effect in the cross-classified model, it was also observed that the random-noise variance was very similar and not significantly different across models. This suggests that the low incidental effects on item responses found for the limited number of sampled incidentals in the cross-classified sample generalize to a larger set of incidental realizations. The highest random-noise variance was observed for the first radical level (cross-classified: 8.036 [7.037, 9.221]; two-level: 7.813 [6.838, 8.963]; difference: 0.223 [−1.298, 1.757]), followed by the second radical level (cross-classified: 6.508 [5.742, 7.405]; two-level: 6.292 [5.552, 7.159]; difference: 0.216 [−0.941, 1.378]) and third radical level (cross-classified: 5.133 [4.496, 5.893]; two-level: 5.365 [4.696, 6.165]; difference: −0.232 [−1.254, 0.779]). The highest level – 1 consistency was observed for the second radical level (cross-classified: 0.459 [0.301, 0.541]; two-level: 0.443 [0.365, 0.520]; difference: 0.015 [−0.155, 0.130]), followed by the first radical level (cross-classified: 0.447 [0.329, 0.530]; two-level: 0.406 [0.325, 0.489]; difference: 0.039 [−0.103, 0.157]) and the third radical level (cross-classified: 0.405 [0.301, 0.490]; two-level: 0.390 [0.304, 0.474]; difference: 0.015 [−0.121, 0.136]).

## General Discussion

In this contribution, we drew attention to the different sampling processes of subjects and item incidentals (surface characteristics of items that are hypothesized to elicit no effects on item parameters) that may be implied across different validation studies of automatic item generators for assessing psychological constructs. In cross-classified sampling, every subject “sees” every incidental realization and incidental effects on the item scores (that should be minimal) can be estimated. Yet, as the number of sampled incidental realizations will often be low due to practical constraints of the data collection, the generalization of sample findings to the complete population of incidental realizations will be limited. In contrast to this, every subject encounters a unique set of sampled incidental realizations in two-level sampling. Here, generalizations of incidental effects are much more comprehensive, but the effects cannot be separated from subject-incidental interactions. Our practical recommendation is to use both designs for an in-depth evaluation of noise stability by comparing the results across the two designs. For that purpose, we introduced a classical test theory AIG model for cross-classified data based on stochastic measurement theory. We showed how a simplification of the model leads to a new model with a two-level sampling process.

In the following, we will first discuss the substantive results of our empirical illustration with respect to the validity of the item generator and potential model extensions. Afterward, we discuss practical limitations that are likely to be encountered in applications, present recommendations for these cases and give an outlook on potential future applications and studies.

### Substantive Conclusions for Figumem and Model Extensions

An application of the models to cross-classified and two-level sampling data for the figural short-term memory test-generator “figumem” (Jendryczko et al., 2020) indeed displayed little effects of the incidentals on the item-score (cross-classified data) and showed that the variance at the subject-incidental interaction level remained stable in a separate sample with a larger set of incidental realizations (two-level data). Hence, results point to considerable noise stability. Yet, level-2 consistency coefficients were not exactly 1 which means that the distinction between incidentals and radicals is not strict. That is, some combinations of frames and emblems (incidental realizations) are more easily recalled than others (see also Jendryczko et al., 2020). However, all level-2 consistency coefficients exceeded .9 pointing to a relatively small impact of incidentals.

To further investigate the importance of incidental realizations, we propose a slightly different study design: One could apply both sampling procedures for incidentals to the same sample of subjects (“two-way incidental sampling approach”). In the case of figumem, for example, a set of nine items (three per radical level) could be sampled for every subject (cross-classified) while for each subject an additional unique set of nine items (again three per radical level) could be sampled (two-level). While the variance decomposition and the investigation of noise stability would remain the same, this would allow for modeling separate variables at the person-level for the cross-classified and two-level items. If correlations among these variables are close to one, this suggests that the construct-relevant additional variability produced by incidentals does not substantially influence the rank-order of subjects and, thus, relative individual ability level.

Interpreting the empirical results with respect to the validity of figumem, we found that the different radical levels (item characteristics that are hypothesized to elicit construct-relevant psychological phenomena and, thus, should elicit differences in item parameters) did not only elicit differences in item difficulties, but also some substantial residual effects in the measurement of the construct. Thus, different radical levels do not only assess increased demands on short-term memory storage capacity, but, apparently, also differential cognitive operations needed to dissociate elements of visual load (different shapes, different types of shape completeness, different shape orientations). Additionally required differential cognitive operations increased with the radical level as the standard radical consistency within the second radical level (one additional operation) was very high (around .9) but decreased substantially (to around .5) within the third radical level (two additional operations). It follows that radicals are not mere determinants of item difficulty and that the item generator is *not* valid with regard to measuring a *single* clearly defined ability variable across all radical levels (although the third radical level seems to be the more problematic one and may hence drive this problem; see also Jendryczko et al., 2020). It is an open question what exactly these different/additional cognitive operations are. Unfortunately, the current data does not provide an answer and the presented models alone cannot answer this question. One approach to overcome this problem could be to include already well-established memory tests and tests that measure memory-adjacent constructs reflecting criterion and discriminant validity. Being imbedded in a flexible multilevel Bayesian SEM framework, the models principally allow to include such additional variables. Moreover, in contrast to simple single-level models, the proposed models separate the subject-level effects from the incidental and incidental-subject interaction effects, allowing to estimate criterion related correlations at the subject level more precisely.

The study is limited in that the samples constitute convenience samples, primarily composed of psychology students, with the cross-classified sample additionally including some students from a second university (see Jendryczko et al., 2020). In future studies, it will be important that subjects are sampled from the same population (or the two-way incidental sampling approach is applied on a single sample directly; see above). Otherwise, it remains unclear if (low) incidental effects found in a cross-classified sample are indeed generalizable to a larger set of incidental realizations in a two-level sample as incidentals might have different effects in different populations. Put another way, if the aim is to generalize from a small sample of incidentals to a larger population of incidental realizations within a specific population of subjects, then the populations of incidentals *and* subjects must remain constant across the two sampling procedures. In the application, we found that the ability variance for the first radical level ( $\sigma_{\eta }^{2}$ ) was considerably lower in the two-level sample. While the credibility interval for the difference across samples contained zero, the lower bound of the interval was much closer to zero than the higher bound which might indicate potential differences in populations.

### Practical Limitations, Options, and Outlook

The proposed models are formulated not only within the framework of stochastic measurement but also within classical test theory. The latter implies a linear relationship between latent and observed variables and constrains their appropriateness when applied to different types of data. In intelligence research, for example, item responses are often ordered categorical or binary (0 = incorrect answer given, 1 = correct answer given) so that linearity will likely be violated. While SEMs for ordered categorical data exist (e.g., Muthén, 1983; Takane & De Leeuw, 1987), one needs to consider that, within these, residuals (such as measurement error or subject-incidental interaction) are determined by remaining model parameters (instead of being freely estimated) and that these models imply different stochastic sampling spaces (e.g., Eid, 1996) which renders a straightforward application of the proposed models questionable.

Many other cognitive ability tests (e.g., figumem) provide count data. Latent Poisson-regression models (Rasch, 1960/1980) are often considered more appropriate for such data, yet, the Poisson distribution comes with its own drawbacks such as the strict assumption of equidispersion (the expectancy equals the variance) and unity of factor loadings. While the Rasch-Poisson-Counts model has been extended to handle item-specific dispersion parameters (Forthmann et al., 2019), free estimation of factor loadings (Myszkowski & Storme, 2021), and both (Beisemann, 2022), the additional modeling of cross-classified or two-level data remains an obstacle. While count data are not continuous in nature, applications of continuous linear models are more defendable due to the wider range of possible item scores—especially when the normality assumption holds. We argue that the proposed models are particularly useful in the domain of cognitive abilities when tests contain figural, numerical, and verbal material for which the manipulation of radicals and incidentals does not imply a change in semantics (e.g., as in verbally formulated statements for personality assessment) and the outcome are counts like in many processing speed (e.g., Doebler & Holling, 2016) and fluency tasks (e.g., Ghanavati et al., 2019).

In addition, the application of continuous response models to ordered categorical data is also common practice and has been defended on a theoretical basis (Robitzsch, 2020). Ordered categories often appear in tests measuring facets of divergent thinking and creativity (e.g., Forthmann et al., 2017) or reading comprehension (e.g., Attali et al., 2022). Besides count data, reaction times that are often used in cognitive aptitude testing (e.g., Doebler & Scheffler, 2016; Kyllonen & Zu, 2016; Wang et al., 2023) are another valid candidate for the application of the models.

In the context of such cognitive ability tests, it should also be mentioned that (AIG) tests are also often constructed to provide proxy measures for general intelligence. The automatic item generation of such tests often relies on a much larger set of radicals (in comparison to figumem), such as many possible combinations of specific rules by which figural stimuli are aligned in visual matrices to form figural analogies (e.g., “MetrixDeveloper” by Freund et al., 2008). In these cases, and in order to determine the impact of radicals on the variance of observed scores, one could also model these as random effects. The proposed models of the current contribution may be modified for including radical effects as random and model fit procedures may be used to evaluate fixed and random radical-effect models against each other.

Promising applications of the models also lie within automatic item generation in the context of personality assessment with large language models (e.g., Götz et al., 2023; Hommel et al., 2022), which often apply continuous response scales (e.g., Bejar, 1977; Kloft & Heck, 2024; Kuhlmann et al., 2017; Simms et al., 2019) or multi-point Likert-scales. However, it might be more difficult in this context to define radicals that have an effect on item difficulty (represented by the intercepts $\mu_{r}$ in our models). Usually, there are specific underlying theories about the “mental” structure of a particular personality dimension. For example, the classic BIG-5 personality trait of conscientiousness is theorized to consist of the facets self-discipline, competence, achievement striving, dutifulness, deliberation, and order (Costa & McCrae, 1992) or of the aspects of industriousness and orderliness (DeYoung et al., 2007). Using radical-specific residual effects at the subject-level (represented by $\alpha_{sr}$ in our models), these different facets/aspects can be represented and generalized to multiple items for each facet/aspect.

Keeping all these things in mind, one also needs to reflect on the general costs and time demands of automatic item generators. These start with the initial efforts for creating the item templates and building the generation engines. These efforts vary depending on the psychological construct and the stimulus material. Many cognitive ability item generators rely on “vague” figural (abstract shapes), numerical (random numbers) or verbal (syllables or words that are not meant to form a semantically meaningful sentence) material. For these, the initial efforts are comparably low and come with comparably little costs as freeware programs (such as R) can be used for template and generation engine creation (e.g., figumem itself; for other examples see Blum & Holling, 2018; Doebler & Holling, 2016; Sun et al., 2019). Yet, other constructs such as personality traits, for example, rely on concrete verbal material with semantically meaningful statements or questions which renders the creation of item templates and item generators much more demanding and cost intensive. One might argue that the current and future developments in artificial intelligence and LLMs in particular may reduce time and costs for the generator-creation phase. Yet, we hypothesize that automatically creating “true” incidentals in semantic verbal material without confounding these with “true” radicals to be much more difficult. Hence, we believe that these item pools will suffer from noise instability which can be detected relying on the presented approaches. If this is the case, each and every item (incidental) will have to be inspected in order to form a pool of noise-stable items producing costs which may outweigh the benefits. Additionally, an automatic on-the-fly item generation will be impossible due to the different impacts of the incidentals on the measures.

## Conclusion

Classical Test Theory-Automatic Item Generation measurement models adhering to the stochastic sampling space of cross-classified and two-level sampling of subjects and incidentals are useful tools for the validation process of automatic item generators as they can be used to investigate noise stability. They are best applied in combination, so that incidental effects can be estimated with cross-classified data and the generalization to a large population of incidentals can be examined with two-level data. While the type of data (categorical, continuous, and count) must be considered before the models are applied, the models are especially promising for the application to item generators for cognitive abilities and might hold potential for generators using large language models.

## Data Availability

The data for this article is open-access and retrievable from https://osf.io/ern58/.

## Note

1. It can be installed from *Github* using the devtools package and the command: devtools::install_github(“Jendryczko/figumem”).

## Appendix

### Appendix A. Prior Specifications for Model Parameters in the Cross-Classified and Two-Level Model

For all variance parameters ( $\sigma_{\eta }^{2}$ , $\sigma_{\alpha_{r}}^{2}$ , $\sigma_{\gamma_{r}}^{2}$ , and $\sigma_{\beta_{r}}^{2}$ in the cross-classified model), we used weakly informative inverse gamma-distributions:

(A1)
$$\sigma^{2}∼\Gamma^{‐1}\left(0.01,0.01\right).$$

For the radical-effect covariance ( $\sigma_{\alpha_{2}\alpha_{3}}$ ), we used a weakly informative normal distribution with a mean of zero and a standard deviation of three:

(A2)
$$\sigma_{\alpha_{2}\alpha_{3}}∼N\left(0,9\right).$$

For each of the intercepts ( $\mu_{1}$ , $\mu_{2}$ , and $\mu_{3}$ ), we used a weakly informative normal distribution with a mean of 10 and a standard deviation of 2. The mean of 10 was chosen because it reflects half of the maximum score. A standard deviation of 2 means that 95% of values fall within the range of 6 to 14.

(A3)
$$\mu_{r}∼N\left(10,4\right).$$

For $\lambda_{2}$ and $\lambda_{3}$ , we used more informative priors. An increase in latent ability as measured with the standard radical level (easiest item) should come with a slightly lower increase in latent ability as measured with a non-standard radical level (more difficult item). Thus, for $\lambda_{2}$ we chose a normal distribution with a mean of 0.9 and a standard deviation of 0.2. For $\lambda_{3}$ we chose a normal distribution with a mean of 0.8 and a standard deviation of 0.2. A lower mean for $\lambda_{3}$ in comparison to $\lambda_{2}$ was chosen because the third radical level is assumed to be the most difficult. For $\lambda_{2}$ , 95% of values fall within the range from 0.5 to 1.3. For $\lambda_{3}$ , 95% of values fall within the range of 0.4 and 1.2.

(A4)
$$\begin{array}{ll} \lambda_{2}∼N\left(0.9,0.04\right), & \lambda_{3}∼N\left(0.8,0.04\right). \end{array}$$
